# Integrative Computational Network Analysis Reveals Site-Specific Mediators of Inflammation in Alzheimer's Disease

**DOI:** 10.3389/fphys.2018.00154

**Published:** 2018-03-02

**Authors:** Srikanth Ravichandran, Alessandro Michelucci, Antonio del Sol

**Affiliations:** ^1^Computational Biology Group, Luxembourg Centre for Systems Biomedicine, University of Luxembourg, Luxembourg, Luxembourg; ^2^NORLUX Neuro-Oncology Laboratory, Department of Oncology, Luxembourg Institute of Health, Luxembourg, Luxembourg; ^3^Luxembourg Centre for Systems Biomedicine, University of Luxembourg, Luxembourg, Luxembourg; ^4^Moscow Institute of Physics and Technology, Dolgoprudny, Russia

**Keywords:** neuroinflammation, Integrative approach, computational modeling, signaling network, sustained inflammatory response

## Abstract

Alzheimer's disease (AD) is a major neurodegenerative disease and is one of the most common cause of dementia in older adults. Among several factors, neuroinflammation is known to play a critical role in the pathogenesis of chronic neurodegenerative diseases. In particular, studies of brains affected by AD show a clear involvement of several inflammatory pathways. Furthermore, depending on the brain regions affected by the disease, the nature and the effect of inflammation can vary. Here, in order to shed more light on distinct and common features of inflammation in different brain regions affected by AD, we employed a computational approach to analyze gene expression data of six site-specific neuronal populations from AD patients. Our network based computational approach is driven by the concept that a sustained inflammatory environment could result in neurotoxicity leading to the disease. Thus, our method aims to infer intracellular signaling pathways/networks that are likely to be constantly activated or inhibited due to persistent inflammatory conditions. The computational analysis identified several inflammatory mediators, such as tumor necrosis factor alpha (TNF-a)-associated pathway, as key upstream receptors/ligands that are likely to transmit sustained inflammatory signals. Further, the analysis revealed that several inflammatory mediators were mainly region specific with few commonalities across different brain regions. Taken together, our results show that our integrative approach aids identification of inflammation-related signaling pathways that could be responsible for the onset or the progression of AD and can be applied to study other neurodegenerative diseases. Furthermore, such computational approaches can enable the translation of clinical omics data toward the development of novel therapeutic strategies for neurodegenerative diseases.

## Introduction

Alzheimer's disease (AD) is one of the most prevalent chronic neurodegenerative disease and is responsible for 60–70% of cases of dementia, thus laying important healthcare problems in countries with aging populations (Burns and Iliffe, 2009). Although the precise cause of the disease is not yet understood, several biochemical and neuropathological studies of brains from individuals with AD provide clear evidences for the involvement of inflammatory pathways (Wyss-Coray and Rogers, 2012). The neurodegenerative processes during the course of the disease essentially render neurons unable to fulfill essential functions, such as signal transmission and network integration in the central nervous system (CNS), thus affecting essential daily activities, such as thinking and moving (Burns and Iliffe, 2009). Importantly, local CNS environment contributes to neurodegeneration and supportive cells, such as glia and endothelial cells, are responsible to maintain an ideal surrounding for neuronal functions (Glass et al., 2010). Several accumulating evidences suggest that neurodegeneration occurs in part because the environment is affected during the disease in a cascade of processes collectively termed neuroinflammation (Morales et al., 2014; Ransohoff, 2016). Sustained or chronic inflammation resulting in neuronal death implies persistence of an inflammatory stimulus or a failure in normal resolution mechanisms. A persistent stimulus may result from environmental factors or due to the formation of endogenous factors, such as protein aggregates, that are perceived by resident immune cells, e.g., microglia, as “stranger” or “danger” signals (Glass et al., 2010). Inflammatory responses that induce autocrine and paracrine neuronal feed-forward/feedback loops as well as influence the neuronal crosstalk with microglia and other CNS cell types may hinder normal resolution mechanisms (Glass et al., 2010; Morales et al., 2014). Although certain inflammatory stimuli are associated to beneficial effects, such as phagocytosis of debris and apoptotic cells where inflammation is linked to beneficial tissue repair processes, uncontrolled and sustained inflammation may result in the production of neurotoxic factors that amplify the underlying disease state (Glass et al., 2010).

The pathological hallmarks of AD in the brain include extracellular amyloid plaques comprising aggregated, cleaved products of the amyloid precursor protein (APP) and intracellular neurofibrillary tangles (NFTs) resulting from hyperphosphorylation of the microtubule-binding protein tau (O'Brien and Wong, 2011). Evidence of an inflammatory response in AD includes changes in microglia morphology—from ramified (resting) to amoeboid (active)—and astrogliosis (manifested by an increase in the number, size, and motility of astrocytes) surrounding the senile plaques (Akiyama et al., 2000; Liang et al., 2008). Elevation of inflammatory factors in culture and animal models are known to typically result in neurodegeneration, and have been reported to be elevated in pathologically vulnerable regions of the AD brain (Wyss-Coray and Rogers, 2012). Several existing genetic, cellular, and molecular changes associated with AD provide clear support for the role of immune and inflammatory processes in the disease (Wyss-Coray and Rogers, 2012).

Omics technologies such as, transcriptomics and proteomics, have enabled the identification of key factors that exhibit differential expression patterns in disease conditions compared to homeostatic states (Dendrou et al., 2016). The related experimental datasets contain rich source of molecular profiles under different disease conditions. In particular, clinical data from patients and age-matched controls offer a wealth of information to be analyzed in order to get insights into the role of specific deregulated processes involved in the disease onset and progression (Dendrou et al., 2016). Due to the recent technological advances, it is now possible to generate and analyze high-throughput data from numerous individuals, even down to the level of single cells (Glass et al., 2010; Ransohoff, 2016). However, the enormous complexity of the molecular and cellular pathways involved in neuroinflammation necessitates parallel implementation of computational analyses to investigate pathophysiological mechanisms across different neurological disorders (Dendrou et al., 2016; Hasin et al., 2017). Means to translate wealth of clinical omics information into practical medical benefit is, however, a fundamental challenge that requires the development and application of novel computational methods for data analyses and interpretation.

In the context of AD, a significant amount of high-throughput omics data can be used to further understand the different deregulated processes that contribute to the disease (Hasin et al., 2017). Importantly, it has been observed that upregulated inflammatory mechanisms co-localize in the AD brain with those regions that exhibit high levels of AD pathology (e.g., frontal neocortex, limbic cortex) and are absent or minimal in brain regions with low AD pathologic susceptibility (e.g., cerebellum) (Akiyama et al., 2000). Furthermore, major efforts have been directed toward the identification of specific molecular players of inflammation and their contribution to the disease, but lack an integrative perspective of common and specific features of inflammation across different brain regions in AD (Morales et al., 2014). For this, datasets obtained by tissue or region-specific molecular profiling of AD post-mortem brain serves as a rich resource to study and analyze the involvement of inflammation in different brain regions affected by AD. In this study, in order to shed more light on neuronal inflammatory signaling pathways associated with AD, we analyzed publicly available gene expression data of six different neuronal populations isolated from post-mortem brains of AD patients. Specifically, the objective of the current study was to identify region-specific signaling pathways likely to be involved in inflammation and how these pathways are distributed across different regions of the brain affected by AD.

## Materials and methods

### Rationale

**Figure 1:**
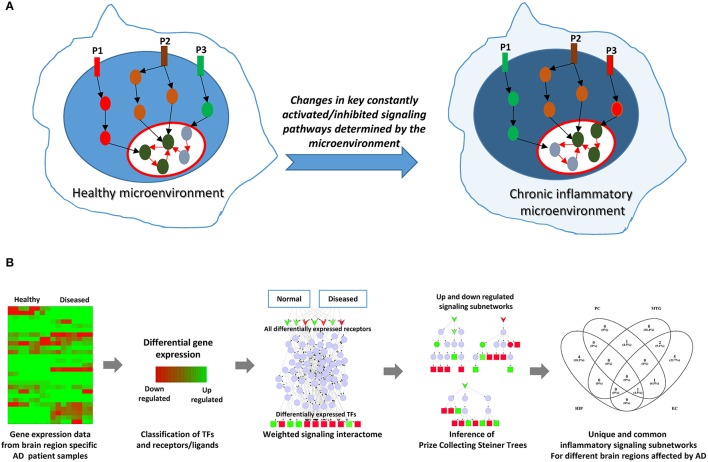
Rationale and the schematic of the computational approach employed. **(A)** Depicts the rationale of the computational network analysis presented in this study. Cellular microenvironment exerts a constant effect on certain key signaling pathways and maintain them constantly active or inactive. Pathways P1, P2, and P3 denote such constantly activated/inhibited signaling pathways under normal healthy conditions and under chronic inflammation. P2 is a pathway commonly active in both conditions. However, P1 is active (upregulated) only in healthy microenvironment while it gets inhibited (downregulated) under chronic inflammation. On the other hand, P3 is activated under chronic inflammation while P1 gets downregulated. The goal in this study is to identify such constantly activated/inhibited signaling pathways as consequence of inflammation in AD affected in different regions of brain. **(B)** Represents the overall schematic if the analysis employed in the study.

Sustained inflammation resulting in tissue pathology can imply persistence of an inflammatory stimulus due to failure in normal clearance mechanisms. A persistent inflammatory stimulus may result from environmental factors or due to the formation of endogenous factors, (e.g., protein aggregates) that eventually cause a sustained activation of certain key intracellular signaling events in the cognate cells, possibly affecting their homeostatic states (Figure 1A). Consequently, certain signaling pathways that were upregulated under normal homeostatic conditions can get downregulated or inhibited under chronic inflammatory conditions, and certain pathways originally inhibited or downregulated under normal conditions can be activated due to chronic inflammation (Figure 1A). In order to infer such constantly activated/inhibited signaling pathways/subnetworks possibly mediated by chronic inflammatory conditions in AD, we adapted a method that we originally developed to identify constantly activated/inhibited signaling subnetworks due to sustained effect of the niche or microenvironment on stem cell state (Ravichandran et al., 2016; Ravichandran and Del Sol, 2017). Briefly, the methodology combines gene expression data with signaling interactome, and identifies sparsest signaling sub-networks by connecting the receptors/ligands with the transcription factors (TFs) that best explain the differential gene expression pattern. For this, we assigned differential weights for interactions (edges) based on expression data and employed a Prize Collecting Steiner Tree (PCST) algorithm to infer minimal subgraphs of the signaling interactome (Figure 1B).

### Gene expression data sources

Since we were interested in studying the role of chronic inflammation in different brain regions affected by AD, we analyzed gene expression datasets obtained from six different neuronal populations located in different areas of the brain. Specifically, neuronal populations from entorhinal cortex, hippocampus, middle temporal gyrus, posterior cingulate cortex, superior frontal gyrus, and primary visual cortex were collected (Liang et al., 2008). Gene expression datasets with appropriate controls are available from the Gene Expression Omnibus (GEO) with Accession No. GSE5281. The samples consisted of 13 control subjects and 10 AD cases for entorhinal cortex, 13 control subjects and 10 AD cases for hippocampus, 12 control subjects and 16 AD cases for middle temporal gyrus, 13 control subjects and 9 AD cases for posterior cingulate, 11 control subjects and 23 AD cases for superior frontal gyrus, and 12 control subjects and 19 AD cases for primary visual cortex (Liang et al., 2008). Further, gene expression was profiled by microarray using Affymetrix Human Genome U133 Plus 2.0 Array platform (Liang et al., 2008).

### Identification and classification of differentially expressed genes

We used the lists of differentially expressed genes (DEGs) directly from the original study (Liang et al., 2008). Supplementary Table 1 lists the DEGs for different neuronal subpopulations used in this study. From the lists of DEGs specific for different brain regions, we identified a set of differentially expressed TFs (DETFs) and transcriptional regulators based on the annotation available at Animal TFDB (http://bioinfo.life.hust.edu.cn/AnimalTFDB/; Hasin et al., 2017). For differentially expressed receptors (DERs), since a complete database of receptor molecules is currently unavailable, we used Gene Ontology classification of receptor activity and plasma membrane (GO:0004872, GO:0005886) to identify DEGs with possible receptor activity. The set of DERs served as the potential sensors of the environment and, specifically, chronic inflammatory stimuli. Further, secreted molecules, such as cytokines and chemokines, can act as regulators of downstream signaling pathways by activating their cognate receptors. In order to infer differentially expressed ligands, we took advantage of the classification of potential ligands reported in a recent study (Ramilowski et al., 2015). For the purpose of our analysis, we discretized the expression data based on differential gene expression and considered genes identified as upregulated and downregulated (based on the above mentioned cutoff) as “1” and “−1”, while the non-DEGs were considered “0.” Supplementary Table 2 contains the classification of the DEGs for different brain regions.

### Compilation of interactions to build a background signaling interactome

In addition to gene expression data, we also required a compiled list of potential signaling interactions with direction (source-target relationship) and sign (activation or inhibition) as an input for our method. For this, we used publicly available signaling interactions databases OmniPath and ReactomeFI (Wu et al., 2010; Turei et al., 2016). We combined them by removing the redundant interactions commonly present in both databases (by removing the duplicate entry) to acquire only unique interactions. We chose these two databases as they are well curated and contain directionality and signs (positive or negative regulation) of signal flow.

### Capturing the effect of chronic inflammatory signals

In order to recapitulate the effect of inflammatory signals on the cells under different diseased conditions, we consider that the upregulated receptors/ligands for the particular disease are under direct influence of the diseased environment (or niche). Since the exact mechanisms of chronic inflammation are not known, we represent them by introducing a dummy inflammation node in the raw signaling network. This dummy node is then connected to all upregulated receptors for each phenotype under consideration. Therefore, signal transduction from the inflammatory niche to DETFs must be propagated through at least one of the upregulated receptors. Such a representation of unknown external influence by a dummy node has been applied earlier (Tuncbag et al., 2013). Therefore, according to our consideration, signal transduction due to inflammation must be propagated through one of the upregulated receptors in order to reach the downstream TFs.

### Calculation of differential edge weights

The edges in the signaling interactome were weighted using the gene expression data. This weighting scheme was implemented in order to maximize the compatibility between the expression data and interaction sign. By compatible, we mean consistency between the sign of the interactions (i.e., positive when activating and negative when inhibiting) and the effect (i.e., activation or inhibition) that the receptor has on its downstream target TFs. For example, sign of a signaling path from a receptor to a TF that is up regulated or overexpressed must be positive (activation), while it must be negative (inhibition) for down regulated or under-expressed TF. We calculated the differential edge weight such that for a given phenotype (for example disease) it reflected the probability of the target gene of the specific interaction to be relatively more active when compared to the other phenotype (for example healthy control) by considering the interaction sign and booleanized expression state of the interacting nodes. For example, considering an interaction A activating B with booleanized expression state of both the nodes being 1 (upregulated) for first phenotype and consequently −1 (downregulated) for the other phenotype. Here, since both nodes are upregulated in the first phenotype, the probability of B being differentially active in first phenotype will be higher in comparison to the second phenotype where both nodes are downregulated. However, when we consider an example of A inhibiting B, the probability of B being differentially active across the two phenotypes is low, and in such cases we consider equally low edge weights for the interaction. Since we worked with booleanized expression values, we considered the fixed probabilities, where an edge was assigned a probability of 0.9 if it was classified as high probability interaction and 0.1 when it was classified as low. Based on such differential edge-weighting scheme, we calculated differential edge weights for the two phenotypes under consideration by accounting for interaction sign and booleanized expression status of the interacting nodes.

### Identification of signaling subnetworks

In this weighted raw signaling interactome, we aimed to identify signaling paths that are potentially affected by chronic inflammation and are responsible for the observed expression pattern of the DETFs. Our method considers that microenvironment maintains the cells in a stable state by a sustained effect on their TFs via constantly activated/inhibited intracellular signaling pathways compatible with the phenotype-specific TRN state. The fact that the cells exhibit differences in their phenotype (diseased vs. healthy) due to differential effect of the microenvironment suggests that the intracellular signaling events controlling the specific GRN for maintain the specific phenotype are also differentially active. For identifying such signaling sub-networks, we employed PCST formalism to infer sub-networks with the dummy inflammation node as the root or origin node and the DETFs as the terminal nodes employing a heuristic algorithm MSGSTEINER (Bailly-Bechet et al., 2011). The Steiner Tree formalism have been used earlier to reconstruct active signaling pathways (Bailly-Bechet et al., 2011). Here, the objective is to infer the sparsest sub-networks that connect the root node (dummy node) and all the terminal nodes (TFs), that is also compatible with the differential expression states of the nodes inferred from the data. Since the dummy node is connected only to the upregulated receptors/ligands, the inferred sub-networks (Steiner trees) will encompass only those receptors that are both topologically favorable and compatible in the expression state to link the DETFs. Therefore, from several hundreds of upregulated receptors/ligands, we could narrow down to the few key ones linking the DETFs based on their unique network topological features. Importantly, as these sub-networks are more topologically favorable to explain the downstream gene expression pattern of TFs, they are likely to represent constantly activated/inhibited signaling sub-networks due to effect of chronic inflammation. In fact, our computational approach attempts to infer the sparsest subnetwork that connects the DERs/ligands and the downstream DETFs, and attempts to include as many differentially expressed intermediates (linker molecules) as possible (by maximizing the edge weights) which are also consistent with the sign of interaction. However, in cases where there are no such intermediates that are differentially expressed, certain non-differentially expressed intermediates are chosen as linker molecules depending on the network topology. Therefore, these intermediates are necessary for signal transduction from the DERs/ligands to DETFs. However, it is important to note that, this does not imply that differential expression of the downstream genes are caused by the intermediates that are not differentially expressed. Further, since we are attempting to capture sustained signaling relying only on gene expression data, some of the molecules that function via post-translational modifications are not differentially expressed but can still act as intermediate linker molecules.

The method infers receptors and associated signaling subnetworks that are crucial for influencing the DETFs in a sustained manner, and does not attempt to rank all the inferred signaling intermediates (other downstream molecules like associated kinases and phosphatases) or the entire pathway as a whole.

## Results

We employed our computational method to identify signaling networks that are likely to be constantly perturbed in AD patients when compared to healthy elderly control individuals. Here, we discuss certain key receptors and their associated signaling components that were identified by our method. Although several of the identified receptors/ligands had a direct link with inflammatory immune responses, the method also inferred molecules not directly related to immune responses, but associated to the disease through other mechanisms. This could be possibly due to the fact that other processes that are responsible of AD progression can also exert sustained influence apart from inflammatory stimuli. Since the method attempts to infer sustained signaling components, irrespective of whether they are derived by inflammatory means or any other process linked to AD, such as deposition of amyloid-beta (Aβ) plaques or neuronal death, it can therefore gather components that are not associated to an inflammatory immune response, but linked to the disease itself. Further, it must be mentioned that we focused predominantly on characterizing the region specific inducers (receptors/ligands) of inflammation and not on all the downstream intermediates and target TFs that transmit the inflammatory signals based on the inferred signaling subnetworks. Table 1 lists the receptors/ligands identified for six different brain regions in the signaling subnetworks with evidences for their involvement in AD focusing on neuronal inflammatory immune responses.

**Table 1:** Identification of AD specific factors.

**Brain region**	**Receptors/ligands identified in the signaling network**			
	**Upregulated**	**Positive role in inflammation/disease**	**Downregulated**	**Negative role in inflammation/disease**
Hippocampus	HMGB1 PPP2CA SHANK3 FGFR1 GRB2	Yes Yang and Tracey, 2010 Yes Rajendran et al., 2017	PTPRF ADAM17 HRAS	Yes Qian et al., 2016
Posterior cingulate cortex	ADAM10 FGFR1 TNFRSF1A	Yes Rajendran et al., 2017 Yes McAlpine and Tansey, 2008	APP PTPRE PTPRD EPHB6 SORT1 PPP2CA	Yes Carlo et al., 2013 Yes Shanley et al., 2001
Middle temporal gyrus	ADAM10 ERBB4 THBS2 ROBO1 EPHB1 NOTCH1 ITPR2 MRC2 EGFR SDC2 INHBB	Yes Song et al., 2012 Yes Coulthard et al., 2012 Yes Siddiqui et al., 2012 Yes Parish, 2006	TUB SORT1 TRAF5 CALR	Yes Carlo et al., 2013 Yes Stemmer et al., 2013
Entorhinal cortex	ERBB3 EGFR PLXNA2 FGFR1 IGF1R FN1 ERBB4 LIFR TNFRSF1A	Yes Siddiqui et al., 2012 Yes Lin et al., 2009 Yes Rajendran et al., 2017 Yes Blazquez et al., 2014 Yes Song et al., 2012 Yes Pan et al., 2008 Yes McAlpine and Tansey, 2008	GNAS LRPAP1 CHRNA7 EPHA4 PTPRR IL12RB2 CHRNB2 GNAI2 TRAF5 PRKCE MST1 GPC1 SHC1	Yes Pandey et al., 2008 Yes Dineley, 2007 Yes Rosenberger et al., 2014
Primary visual cortex	EGFR FGFR1 ROBO1	Yes Siddiqui et al., 2012 Yes Rajendran et al., 2017	CNTN1 PPP2CA ERBB4	Yes Shanley et al., 2001
Superior frontal gyrus	EGFR	Yes Siddiqui et al., 2012	PPP2CA BDNF	Yes Shanley et al., 2001 Yes Jiao et al., 2016

In the original study, the analysis was conducted comparing non-tangle-bearing neurons from AD patients with healthy neurons from control elderly subjects. In fact, the brain regions analyzed have been previously observed to show characteristic pathological differences in the brains of individuals afflicted with AD compared to healthy individuals. The entorhinal cortex and the hippocampus are two regions that have been found to be susceptible to early NFT formation (Bouras et al., 1994). The mid temporal gyrus and the posterior cingulate cortex have been found to exhibit an elevated susceptibility to amyloid plaque formation (Blesa et al., 1996). The superior frontal gyrus has been observed to show metabolic changes relative to normal aging (Blesa et al., 1996), and the primary visual cortex has been found to be relatively unaffected from any form of age-related or disease-related neurodegeneration (Liang et al., 2008). These two regions are also known to represent late stages of AD (Liang et al., 2008).

### Hippocampus

The hippocampus is critical for learning and memory, is specifically vulnerable to damage at early stages of AD (Mu and Gage, 2011). In fact, perturbed neurogenesis in the adult hippocampus indicates an early critical event in the onset and progression of AD. From a functional point of view, hippocampal neurogenesis plays an important role in structural plasticity and network maintenance (Mu and Gage, 2011). The CA1 region was selected in the original study (from where the gene expression data for the computational analysis was obtained) because this area is the earliest (Braak stages I–IV) and most heavily affected region of the hippocampus in terms of tangle formation (Liang et al., 2008).

**Figure 2:**
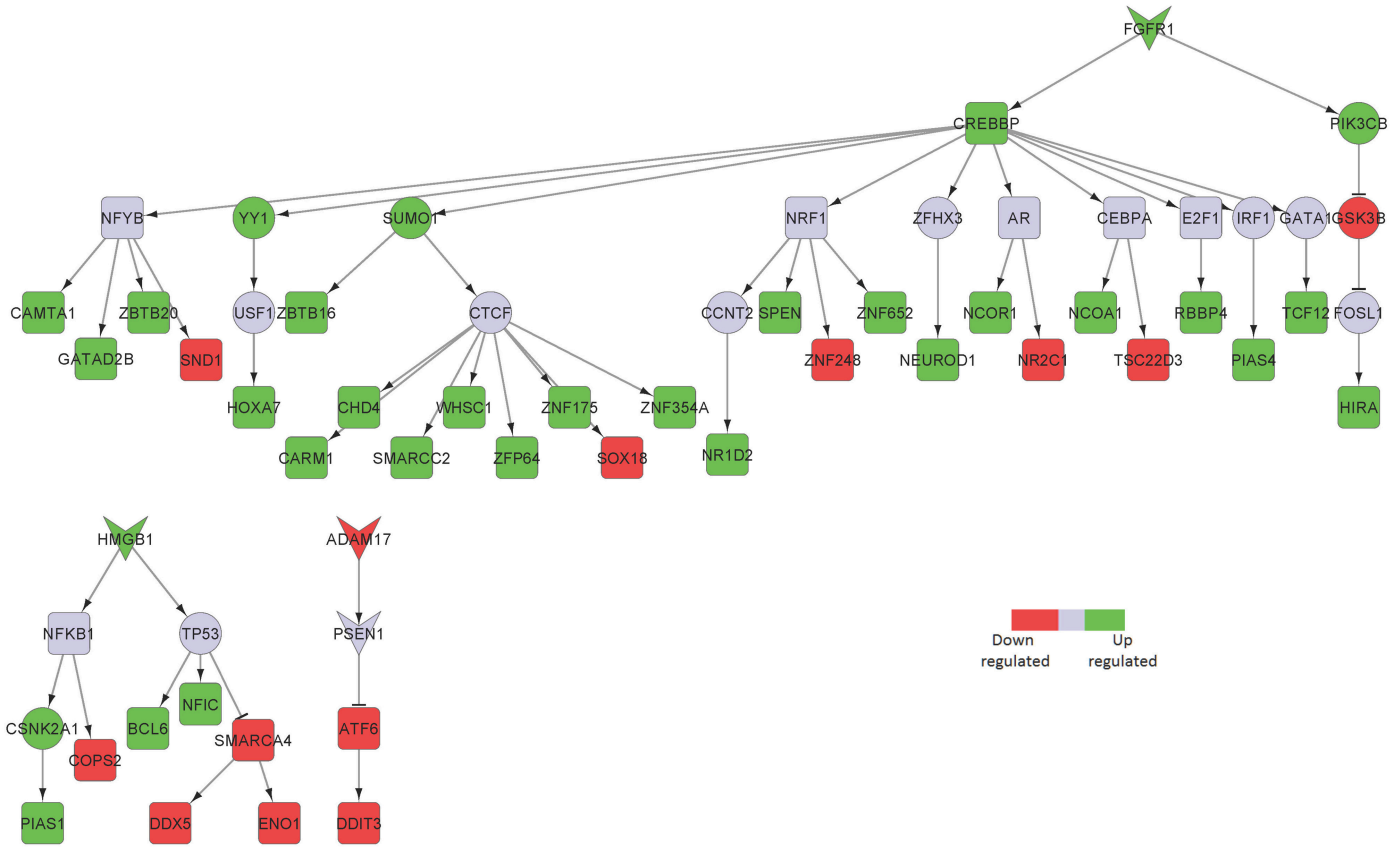
Key signaling networks identified for the hippocampus. The figure shows three key signaling subnetworks controlled by FGFR1, HMGB1, and ADAM17 identified for hippocampus with known role in AD. FGFR1 mediated subnetwork was found to control of 23 up- and 6 downregulated TFs, HMGB1 mediated pathways controlled 3 upregulated and 4 downregulated TFs, and ADAM17 controlled 2 downregulated TFs. Further, FGFR1 and HMGB1 represent constantly activated signaling subnetworks while ADAM17 represent constantly inhibited signaling pathways. Inverted triangles denote receptor/ligand molecules, circular nodes represent signaling intermediates, and square nodes represent transcription factors. Any node that is not a TF or a receptor/ligand is considered a signaling intermediate such as kinases/phosphatases and adapter molecules. The edges with arrowhead denote activation and those with dashed head denote inhibitory interactions. Green nodes indicate upregulated genes in hippocampus region of AD brain, while the red nodes indicate downregulated ones. Blue nodes represent those intermediates that are not differentially expressed but serve to link the differentially expressed receptor/ligand to the downstream TFs. Supplementary Figure 1 contains the other subnetworks identified for hippocampus that did not have known role in AD.

We identified fibroblast growth factor receptor 1 (FGFR1) mediated signaling activity as a key upregulated component in neurons isolated from the hippocampus of AD patients (Figure 2). FGFR1 signaling is known to transmit inflammatory signals through regulation of other surface proteins (Woodbury and Ikezu, 2014). In fact, due to its importance in adult neurogenesis and neuroinflammation, manipulation of fibroblast growth factor 2 (FGF2)/FGFR1 signaling has been a focus of therapeutic development for neurodegenerative disorders, such as AD, multiple sclerosis (MS), Parkinson's disease and traumatic brain injury (Woodbury and Ikezu, 2014). In our sub-network, FGFR1 signals through CREB binding protein (CREBBP) induction, a protein involved in the transcriptional co-activation of many different TFs, such as the inflammatory mediators interferon regulatory factor 1 (IRF1) and E2F transcription factor 1 (E2F1), the latest involved in the modulation of neuronal apoptosis (Hou et al., 2000). High mobility group box 1 (HMGB1) is a mediator of inflammation that is released extracellularly during cells death or secreted by activated cells (Jiao et al., 2016). In line with its involvement in inflammatory processes, HMGB1 upregulation in our sub-network is linked to tumor protein p53 (TP53) and nuclear factor kappa b subunit 1 (NFKB1), two main inflammatory signaling pathways.

Among the downregulated receptors, ADAM metallopeptidase domain 17 (ADAM17), a metalloprotease involved in the processing of tumor necrosis factor alpha (TNF) that has been described to counteract inflammation and further neuronal damage, was identified (Figure 2; Qian et al., 2016).

### Posterior cingulate cortex

The precise function of the posterior cingulate cortex is not yet clearly established. However, it is known to be involved in cognitive tasks (Leech et al., 2012). This region is known to acquire early amyloid deposition, reduced metabolism in AD (Leech and Sharp, 2014), and therefore serves as a key region to be studied despite the lack of clarity in its functionality in the brain.

**Figure 3:**
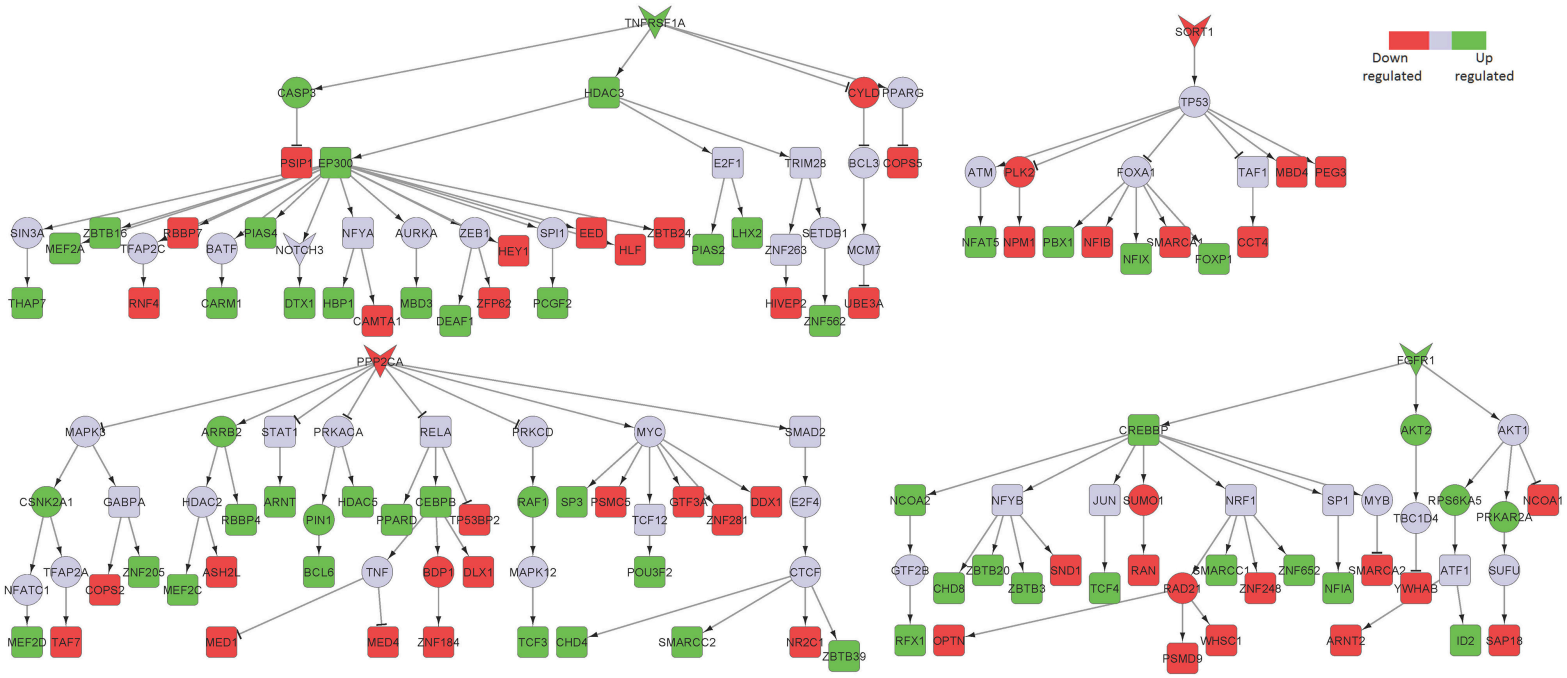
Key signaling networks identified for the posterior cingulate cortex. The figure shows four key signaling subnetworks controlled by TNFRSF1A, FGFR1, SORT1, and PPP2CA identified for posterior cingulate cortex with known role in AD. TNFRSF1A mediated subnetwork consisted of 15 up and 12 down regulated TFs, FGFR1 mediated subnetwork consisted of 8 up- and 6 downregulated TFs, SORT1 mediated subnetwork consisted of 4 up- and 6 downregulated TFs and PPP2CA mediated subnetwork consisted of 15 up- and 13 downregulated TFs. TNFRSF1A and FGFR1 controlled subnetworks represent constantly activated signaling subnetworks while SORT1 and PPP2CA subnetworks represent constantly inhibited ones. The figure legends are the same as that in Figure 2. Supplementary Figure 2 contains the other subnetworks identified for posterior cingulate cortex that did not have known role in AD.

TNF receptor superfamily member 1 (TNFRSF1A), a known pro-inflammatory signaling component, was a major player in the posterior cingulate cortex sub-network (Figure 3). Excess of inflammatory mediators in the brain are associated, at least partly, to activated microglia, which accumulate around amyloid-beta (Aβ) plaques in AD brains, showing chronic activation and therefore signaling constantly. Elevated levels of pro-inflammatory cytokines, such as TNF, could potentially inhibit phagocytosis of Aβ in AD brains thereby hindering efficient plaque removal by resident microglia (McAlpine and Tansey, 2008). Moreover, FGFR1, also upregulated in the hippocampal neurons from AD patients, was identified in the posterior cingulate cortex region.

Among the downregulated signaling networks, we identified sortilin 1 (SORT1), a pro-neurotrophin receptor which plays a major role in the clearance of apolipoprotein E (APOE)/Aβ complexes in neurons (Figure 3; Carlo et al., 2013). APOE sequesters neurotoxic Aβ peptides and deliver them for cellular catabolism via neuronal APOE receptors (Carlo et al., 2013). SORT1 binds APOE with high affinity and lack of receptor expression in mice results in accumulation of APOE and Aβ in the brain and in aggravated plaque burden, thus suggesting a link between Aβ catabolism and pro-neurotrophin signaling converging to this receptor (Carlo et al., 2013). Another receptor identified in the network was protein phosphatase 2 catalytic subunit alpha (PPP2CA). It binds to tau and is the primary tau phosphatase (Sontag and Sontag, 2014). Its deregulation correlates with increased tau phosphorylation likely contributing to tau deregulation in AD (Sontag and Sontag, 2014). The signaling network controlled by SORT1 was also inferred in the middle temporal gyrus network, while PPP2CA was inferred in the superior frontal gyrus network (Table 1), thereby implicating their role in other affected brain regions.

### Middle temporal gyrus

This brain region is known to be involved in cognitive processes including, language and semantic memory processing, visual perception, and multimodal sensory integration (Onitsuka et al., 2004). Further, the middle temporal gyrus is known to exhibit reduced metabolic activity in AD (Liang et al., 2008).

**Figure 4:**
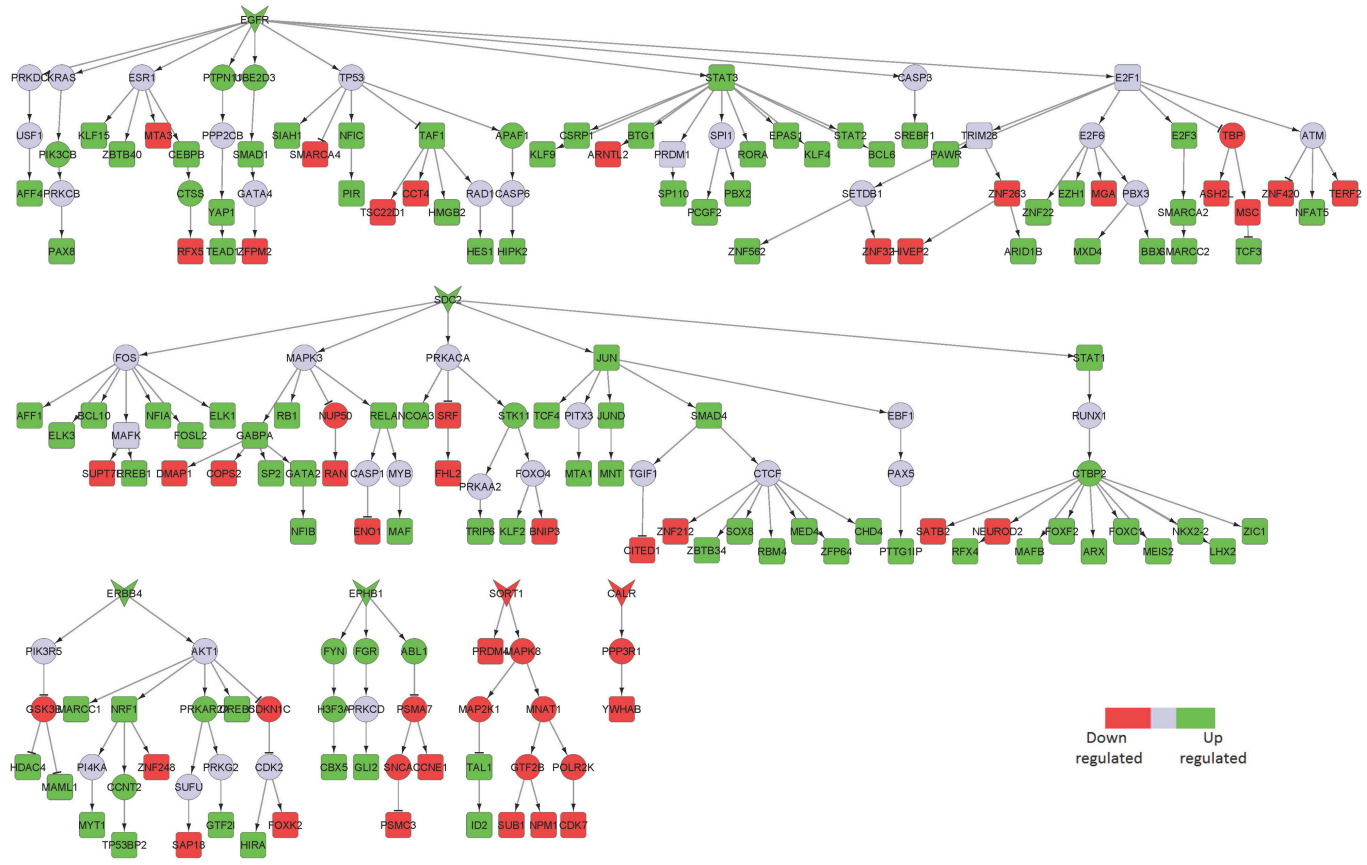
Key signaling networks identified for the middle temporal gyrus. The figure shows five key signaling subnetworks controlled by EGFR, SDC2, ERBB4, EPHB1, SORT1, and CALR for middle temporal gyrus with known role in AD. EGFR controlled subnetwork consisted of 39 up- and 15 downregulated TFs, SDC2 subnetwork consisted of 39 up- and 12 downregulated TFs, ERBB4 subnetwork consisted of 9 up- and 3 downregulated TFs, EPHB1 subnetwork consisted of 2 up- and 2 downregulated TFs, SORT1 consisted of 2 up- and 4 downregulated TFs, CALR consisted of one downregulated TF. Further, subnetworks controlled by EGFR, SDC2, ERBB4, EPHB1 represent constantly activated signaling while the ones controlled by SORT1 and CALR represent constantly inhibited signaling pathways. The figure legends are the same as that in Figure 2. Supplementary Figure 3 contains the other subnetworks identified for middle temporal gyrus that did not have known role in AD.

Syndecan 2 (SDC2) and ERB-B2 receptor tyrosine kinase 4 (ERRB4) mediated signaling were identified in our sub-networks to play key roles in the middle temporal gyrus area (Table 1; Figure 4). In addition, we found Notch1 mediated signaling to be crucial in that region. Notch1 signaling is essential for various CNS functioning from brain development to adult brain function (Brai et al., 2016). Reduction in Notch1 expression affects synaptic plasticity, memory and olfaction. On the contrary, Notch1 over-activation after brain injury is detrimental for neuronal survival (Brai et al., 2016). Some familial AD mutations in presenilins can affect Notch1 processing/activation (Brai et al., 2016). Further reports described Notch1 overexpression in sporadic AD.

In the downregulated signaling networks, calreticulin (CALR) and its downstream network was identified by our method (Figure 4). Calreticulin is found in a complex with APP and Aβ and levels of the calreticulin mRNA and protein are reduced in patients with AD. This suggests that calreticulin is implicated in the proteolytic processing of APP and, thus, in AD pathogenesis (Stemmer et al., 2013).

### Entorhinal cortex

The entorhinal cortex is thought to be a major input and output structure of the hippocampal formation, acting as the nodal point of cortico-hippocampal circuits (Canto et al., 2008). This is one of the most vulnerable brain regions that is attacked during the early stages of AD (Van Hoesen et al., 1991) and is thought to spread from here to other regions of brain. Further, there are emerging roles of inflammation in promoting neurodegeneration in the entorhinal cortex (Criscuolo et al., 2017).

**Figure 5:**
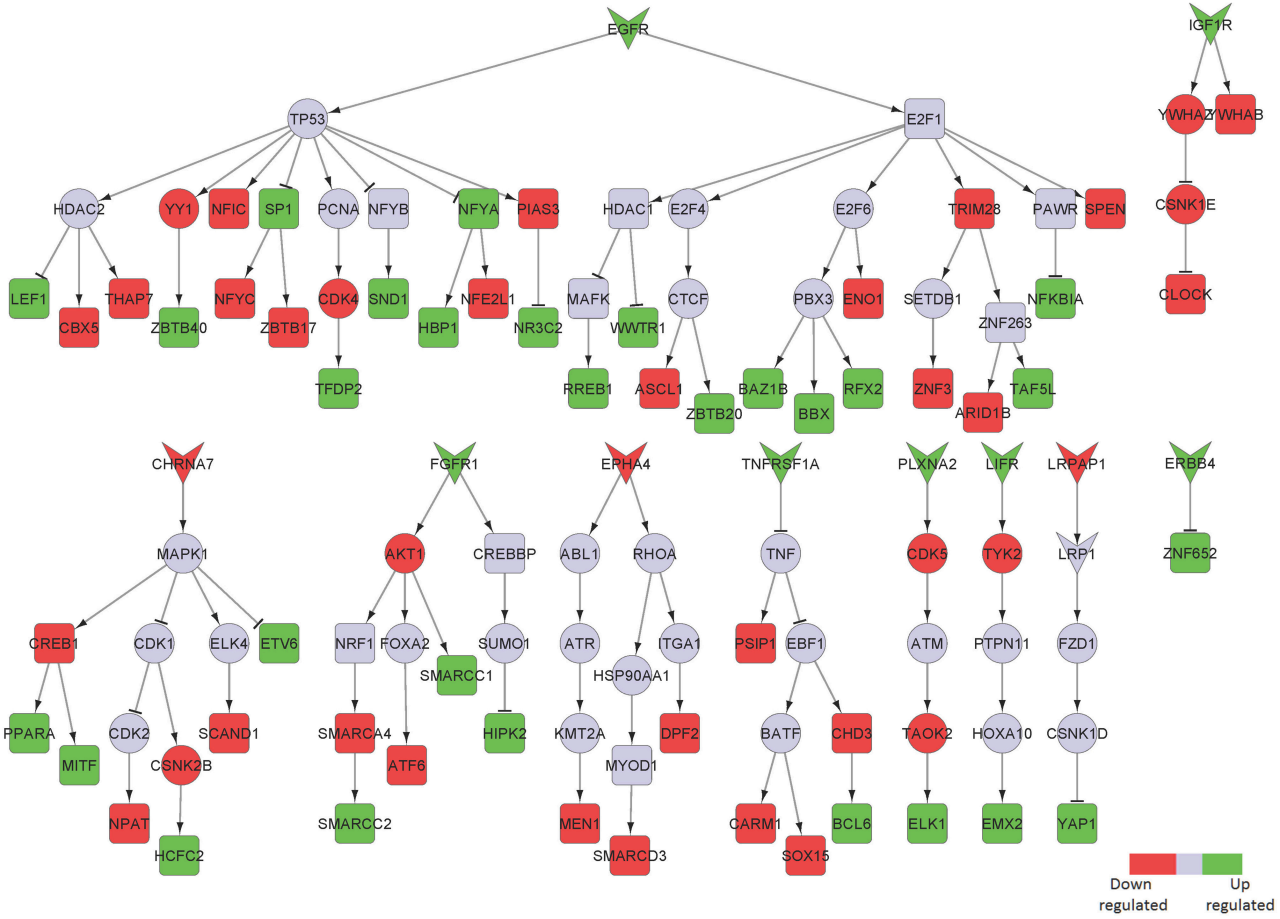
Key signaling networks identified for the entorhinal cortex. The figure shows ten key signaling subnetworks controlled by EGFR, IGF1R, CHRNA7, FGFR1, EPHA4, TNFRSF1A, PLXNA2, LIFR, LRPAP1, and ERRB4 identified for entorhinal cortex with known role in AD. The signaling subnetworks controlled by EGFR, IFG1R, FGFR1, TNFRS1A, PLZNA2, LIFR, and ERBB4 represent constantly activated signaling, while the ones controlled by CHRNA7, EPHA4, and LRPAP1 represent constantly inhibited ones. Further, EGFR subnetwork was found regulate most of the differentially expressed TFs by controlling 16 upregulated and 12 downregulated TFs. The figure legends are the same as that in Figure 2. Supplementary Figure 4 contains the other subnetworks identified for entorhinal cortex that did not have known role in AD.

Epidermal growth factor receptor (EGFR)-mediated signaling was identified as a key upregulated component of the entorhinal cortical neurons isolated from AD patients compared to healthy subjects (Figure 5). Although EGFR is not directly implicated in neuroinflammation, it is known to play a central role in neurometabolic aging. EGFR acts as a signaling entity for several ligand mediated mechanisms and cellular stress responses directly related to aging and degeneration (Siddiqui et al., 2012). Further, EGFR signaling has been implicated in a spectrum of neurometabolic conditions, such as metabolic syndrome, diabetes, AD, cancer, and cardiorespiratory function (Siddiqui et al., 2012). More recently, it has been observed that inhibition of EGFR enables rescue of memory loss in both mouse and drosophila.

LDL receptor related protein associated protein 1 (LRPAP1) and its downstream signaling network was found to be downregulated in this brain region (Figure 5). Interestingly, LRPAP1 levels have been found to be low in patients with increased susceptibility to AD, which implicates a link of this receptor with Aβ clearance. Co-localization of Aβ, APOE and LRPAP1 on senile plaques suggests its involvement in the clearance of APOE/Aβ complex (Pandey et al., 2008). In recent years, the lipoprotein receptor low-density lipoprotein receptor-related protein 1 (LRP1), the down-stream effector of LRPAP1 in our sub-network, emerged as an important regulator of the inflammatory response (May, 2013).

### Primary visual cortex and superior frontal gyrus

**Figure 6:**
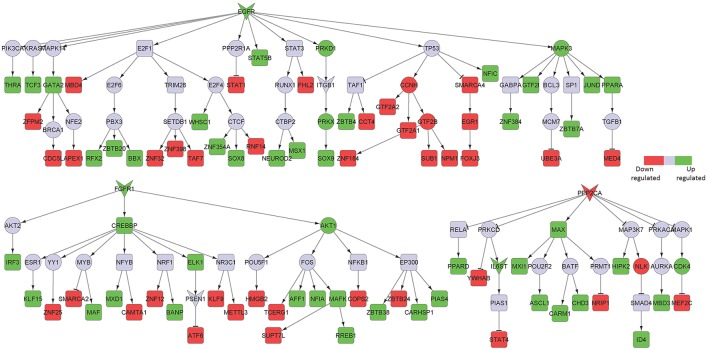
Key signaling networks identified for the primary visual cortex. The figure shows three signaling subnetworks controlled by EGFR, FGFR, and PPP2CA for the primary visual cortex with known role in AD. EGFR signaling subnetwork was found to regulate 20 up- and 21 downregulated TFs, FGFR signaling subnetwork was found to regulate 13 up- and 12 downregulated TFs, and PPP2CA signaling subnetwork was found to regulate 9 up- and 4 downregulated TFs. Further, EGFR and FGFR1 subnetworks were found to be constantly activated while PPP2CA signaling subnetwork was constantly inhibited. The figure legends are the same as that in Figure 2. Supplementary Figure 5 contains the other subnetworks identified for primary visual cortex that did not have known role in AD.

The primary function of the early visual cortex is visual perception (Petro et al., 2017) and the superior frontal gyrus is thought to contribute to higher cognitive functions, in particularly to working memory (du Boisgueheneuc et al., 2006). These two brain regions have been included in the original study as they are known to be later (Braak stages V and VI) and less affected by the disease, and consist of least number of DEGs among the analyzed brain regions (Supplementary Table 1). The major signaling pathway upregulated in these two regions involved EGFR in both areas and FGFR1 in the primary visual cortex. These receptors were also identified by our analysis in other affected brain regions (Table 1; Figure 6).

**Figure 7:**
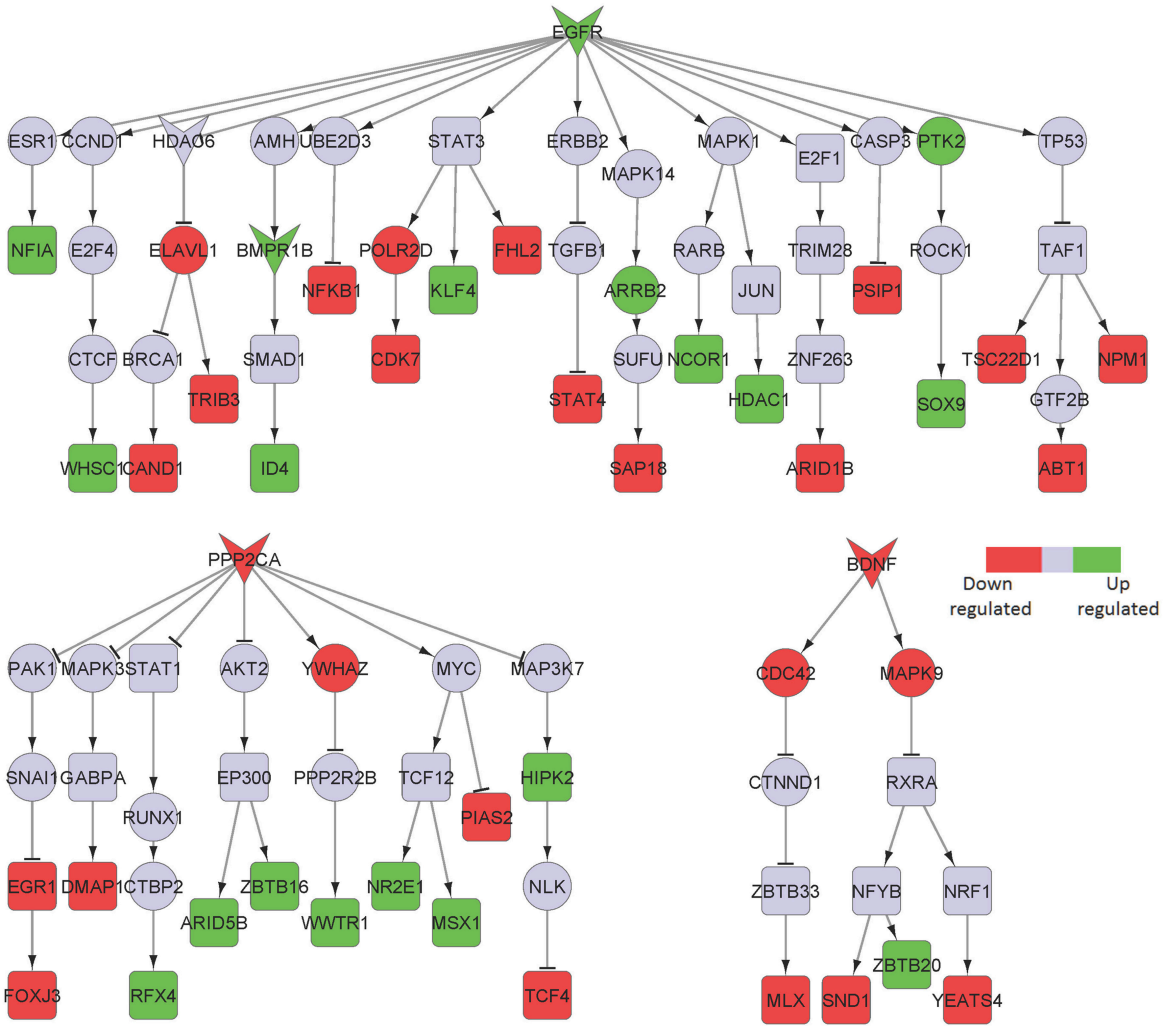
Key signaling networks identified for the superior frontal gyrus. The figure shows three signaling subnetworks controlled by EGFR, PPP2CA, and BDNF for the superior frontal gyrus with known role in AD. EGFR signaling subnetwork was found to be constantly activated and controlled 7 up- and 11 downregulated TFs. PPP2CA and BDNF were found to be constantly inhibited signaling subnetworks. PPP2CA was found to regulate 7 up- and 5 downregulated TFs, while BDNF was found to regulate 1 up- and 3 downregulated TFs. The figure legends are the same as that in Figure 2.

Among the downregulated effectors, the phosphatase PPP2CA, already identified as decreased in the posterior cingulate cortex and upregulated in the hippocampus, was identified in both areas. Furthermore, we found brain-derived neurotrophic factor (BDNF) and its downstream signaling to be specifically downregulated in the superior frontal gyrus area (Figure 7). Importantly, BDNF is known to protect against tau-related neurodegeneration in Alzheimer's disease (Jiao et al., 2016).

### Brain regions sharing common inflammatory mediators

**Figure 8:**
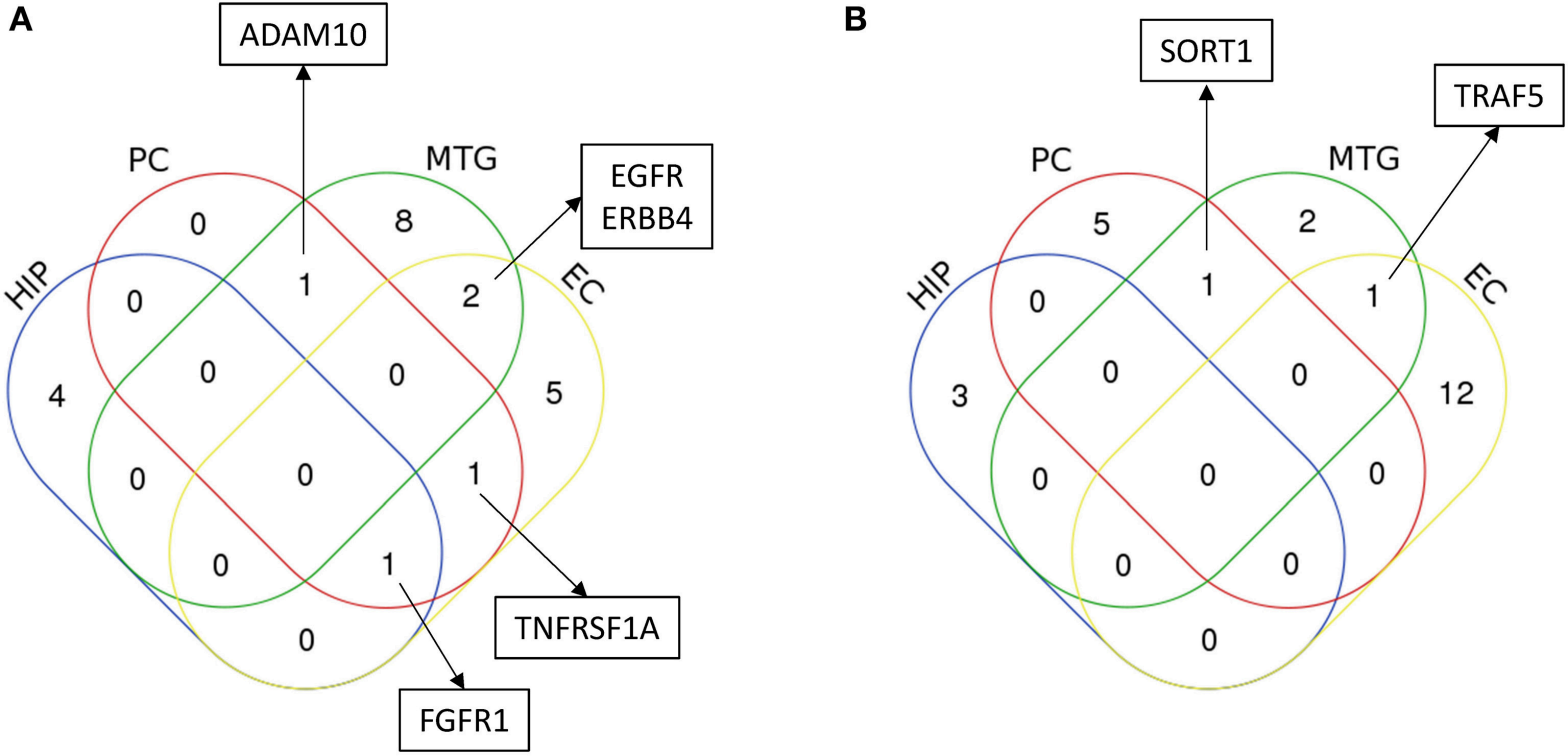
Characterization of common and unique region specific inflammatory regulators in AD brain inferred from the computational analysis. The figure shows the Venn diagram comparing the inferred regulators of inflammation **(A)** upregulated in AD, and **(B)** downregulated in AD, for four major regions AD patients analyzed in this study. These regions are hippocampus (HIP), posterior cingulate cortex (PC), middle temporal gyrus (MTG) and entorhinal cortex (EC). Regulators (only considering receptors/ligands) found to be important for more than one brain regions are highlighted in the box. In addition, in the case of upregulated signaling subnetworks, EGFR signaling was identified for primary visual cortex and superior frontal gyrus in addition to its role in middle temporal gyrus and entorhinal cortex, and FGFR1 signaling was identified for primary visual cortex. In the case of downregulated signaling subnetworks, PPP2CA was found to be commonly present in primary visual cortex and superior frontal gyrus.

After identifying the signaling sub-networks likely to be constantly activated/inhibited in different neuronal subpopulations of AD affected brain, we identified the common and specific factors among different brain regions. As it could be seen from Figure 8, only few identified factors were shared across different brain regions, while the majority of the factors were specific for the respective brain region. Notably, EGFR signaling was identified to be active for four regions, namely, middle temporal gyrus, entorhinal cortex, primary visual cortex and superior frontal gyrus. In fact, recent studies in AD mouse models have observed that EGFR is a preferred target for treating Aβ-induced memory loss, adding value to our computational inference (Wang et al., 2012). Another key receptor found to be commonly active in posterior cingulate cortex and entorhinal cortex is TNFRSF1A, which is a well-known pro-inflammatory factor (Carlo et al., 2013). More importantly, blocking TFN signaling either via genetic manipulation or using chemical inhibitors, reduced the accumulation of intraneuronal amyloid-associated proteins triggered by chronic systemic inflammation, and could possibly act as a valid therapeutic target to modify disease progression during the early stages of AD (McAlpine et al., 2009). FGFR1 mediated signaling pathway, which is known to have profound roles in neurogenesis, was found to be present in entorhinal cortex, hippocampus and primary visual cortex (Woodbury and Ikezu, 2014). Further, activation of this signaling by FGF2 has proven to be highly efficient for the regeneration of neurons in multiple experimental animal models (Woodbury and Ikezu, 2014). In fact, several groups have shown the potential use of FGF2 as a therapeutic for neurodegenerative conditions including AD and PD. FGF2 gene transfer in AD transgenic mouse models is known to significantly restore spatial learning, hippocampal CA1 long-term potentiation, and neurogenesis in the SGZ (Kiyota et al., 2011). Another active signaling pathway commonly present in middle temporal gyrus and primary visual cortex were mediated by ADAM10 and this signaling has known roles in AD (Yuan et al., 2017).

In the case of inhibited signaling pathways (Table 1), only SORT1 and PPP2CA were identified to be commonly present in multiple brain regions. SORT1 was present in the middle temporal gyrus and primary visual cortex, and this molecule has recently been found to act as a novel receptor for apolipoprotein E (APOE) (Carlo, 2013; Carlo et al., 2013). Importantly, ablation of sortilin expression in mice results in accumulation of APOE and Aβ in the brain resulting in AD like physiology the mice (Carlo, 2013). PPP2CA was another key inhibited signaling identified to be present in three different brain regions namely, posterior cingulate cortex, primary visual cortex and superior frontal gyrus. Alterations in this phosphatase activity have been reported in AD-affected brain regions and has been linked to tau hyperphosphorylation, amyloidogenesis and synaptic deficits (Sontag and Sontag, 2014).

## Discussion

Technical advancements in sequencing allow the analysis of thousands of molecular profiles from clinical samples with high quality. These high-throughput techniques open-up opportunities for the development of computational analysis tools to infer meaningful patterns from big data. In this study, we have analyzed gene expression profiling data of specific neuronal populations collected by laser capture microdissection from postmortem samples of AD patients with the aim of identifying brain region specific signaling subnetworks affected by chronic inflammation. The network analysis we carried out enabled the identification of a fraction of receptor/ligands (and the associated downstream signaling networks) as key inflammatory mediators from a large number of DERs and ligands. In fact, on average, there were about 100 DERs/ligand molecules in the datasets we analyzed, and our method was able to identify crucial inflammation mediators. This highlights the utility of network approaches to refine and extract accurate and relevant information from high-throughput datasets. Furthermore, computational analyses are amenable to consider more than one diseased region simultaneously and therefore can be used to evaluate the commonalities and distinct features of regulatory processes involved in different regions in an integrative manner. In addition, when data are available, approaches like ours can be employed to study differences and commonalities of a specific disease phenotype in the context of different regions or tissues that are affected due the disease.

EGFR is one of the key molecule identified from our analysis to be common for four different regions namely, middle temporal gyrus, entorhinal cortex, primary visual cortex and superior frontal cortex of AD brain. Importantly, recent evidences suggest a crucial role for EGFR in AD pathogenesis, where potential interactions between Aβ oligomers and EGFR were found (Wang et al., 2012). Furthermore, inhibition of EGFR led to reversal of memory loss in AD mouse models (Wang et al., 2012). Notably, brain regions known to be less affected by AD, such as primary visual cortex and superior frontal gyrus, contained lesser number of sub-networks compared to regions highly perturbed by the disease, such as entorhinal cortex and middle temporal gyrus. This probably suggests that changes in AD brains are more pronounced in the main affected areas when compared to less influenced regions. This feature could also be partly attributed to the less number of DEGs in these two regions of AD brain (Supplementary Table 1). Despite the lower number of DEGs in the primary visual cortex and the superior frontal gyrus, EGFR was found to be crucial in these two regions in addition to its role in the middle temporal gyrus and the entorhinal cortex. This could also possibly reflect a crucial role for EGFR in the progression of AD in both early and late stages of the disease. It has to be mentioned that EGFR was not upregulated in two other important regions, i.e., the hippocampus and the posterior cingulate cortex at least in the dataset we analyzed. It could possibly reflect region specific perturbation of key molecules and it also might relate to the fact that EGFR can also be regulated post-translationally and not always at the gene expression level.

Our computational analysis of AD samples from six different brain regions identified certain common regulators, such as FGFR1 and PPP2CA, known to be key neuroinflammatory factors (Tuncbag et al., 2013; Rajendran et al., 2017). In addition, certain region specific factors, such as CALR and EPHB1 in the middle temporal gyrus, were also identified by our analysis. In addition to several receptor/ligand molecules with direct role in inflammation, our analysis identified several novel candidates whose direct link to inflammation and the disease is not clearly known. For instance, we identified the Notch receptor, NOTCH1, in the middle temporal gyrus network. Although this receptor is not directly related to neuroinflammation, the role of Notch signaling pathway in promoting pro-inflammatory responses in the cells expressing NOTCH1 has been observed (Brai et al., 2016). Such molecules with no direct evidences in inflammation are novel predictions from our analysis, and will be interesting to experimentally test if they are important to transmit inflammatory signaling in these disease conditions.

Overall, our analysis revealed mostly distinct and few common inflammatory signaling components across the different neuronal populations analyzed. However, despite the predominance of region specific factors, the analysis identified certain shared inflammatory factors, reflecting some environmental similarities across the affected brain regions, such as the presence of Aβ plaques. Further, we observed that not *all* receptors/ligands that were identified by our analyses were directly related to inflammation, but were known to be implicated in the disease via some other processes, such as metabolic dysregulation (for example, SORT1). This is because, although we considered inflammation to be one of the predominant cause of sustained signaling activities, our computational approach infers constantly activated/inhibited signaling pathways irrespective of whether these pathways are induced by inflammation or any other cellular processes. Although this aspect could represent a limitation of our approach, it rather demonstrates that it can be applied in cases where sustained signaling pathways need to be identified (Ravichandran et al., 2016; Ravichandran and Del Sol, 2017). Further, certain classical inflammatory mediators, such as FAS- and IL6-related pathways, were not identified. This could be explained by the fact that our approach requires that the modulation of the factors has to occur at gene expression level, and that our approach does not take into account specific receptors and TFs that are not differentially expressed under the disease condition. However, these mediators can still be active during inflammatory processes, for instance via post translation modifications, and our approach cannot detect such mediators since it currently relies only on mRNA level changes. In general, these limitations can be addressed when we consider proteomics or phosphoproteomics datasets for the inference of sustained signaling. However, availability of such protein level datasets are still limited compared to transcriptomics datasets and despite such limitations, predominantly due to incomplete data, we observed that our computational approach revealed the involvement of several disease related pro- and anti- inflammatory factors highlighting the value of such analysis.

To our knowledge, this is one of the first study, which computationally analyzed the potential implication of chronic inflammation on AD to infer the induced signaling networks/pathways. In this regard, it must be mentioned that two recent studies have attempted to infer common and unique pathways/signatures in different neurodegenerative diseases (Li et al., 2014, 2015). However, their focus was more on “generic” pathways affected due to the disease and not specifically related to inflammation or sustained signaling. Further, in both studies, the analysis was predominantly based on differential gene expression signatures and pathway enrichment, and did not attempt to capture dysregulated network components as described in our study (Li et al., 2014, 2015). Although these studies are very useful to understand the overall pathways that are dysregulated in the disease condition and to define disease signatures, they do not capture specific molecular features that can relate the pathways to the disease itself. Alternatively, our analysis focus on inference of sustained signaling subnetworks to capture specific molecular features of chronic inflammation, which was not the focus of the other studies. Consequently, our current analysis revealed specific factors that were up- or down- regulated in neuronal populations from different brain regions affected by AD that were implicated in inflammation and the disease.

In the context of neurodegenerative diseases, integrative approaches enable to obtain a holistic understanding of the processes and factors that initiate and sustain specific disease pathologies that act primarily at the cellular level. For instance, protective and pathogenic roles of glial cells, such as microglia and astrocytes, in addition to the activation of common inflammatory pathways in these cells in several neurodegenerative diseases, support the concept that glia-induced inflammation can possibly sustain the disease pathology (Burns and Iliffe, 2009; Wyss-Coray and Rogers, 2012). In this context, integrative computational approaches like ours enable the identification of factors whose perturbation (by either activating or inhibiting) could reduce the production of factors that contribute to neurotoxicity, thereby potentially resulting in clinical benefit in specific neurodegenerative diseases.

## Author contributions

SR and AdS conceived the idea. SR performed the computational analysis. All authors contributed to the writing of the manuscript and approved the final version of the manuscript. AdS coordinated the overall project.

### Conflict of interest statement

The authors declare that the research was conducted in the absence of any commercial or financial relationships that could be construed as a potential conflict of interest. The reviewer JEE and handling Editor declared their shared affiliation.
