# Supplementary material for: Integrative Computational Network Analysis Reveals Site-Specific Mediators of Inflammation in Alzheimer's Disease
Source: Front Physiol. 2018 Mar 2;9:154. doi: 10.3389/fphys.2018.00154 (PMC5840953; doi:10.3389/fphys.2018.00154)
Supplement: Supplementary file 3 [file Image1.pdf]

Supplementary Figure 1

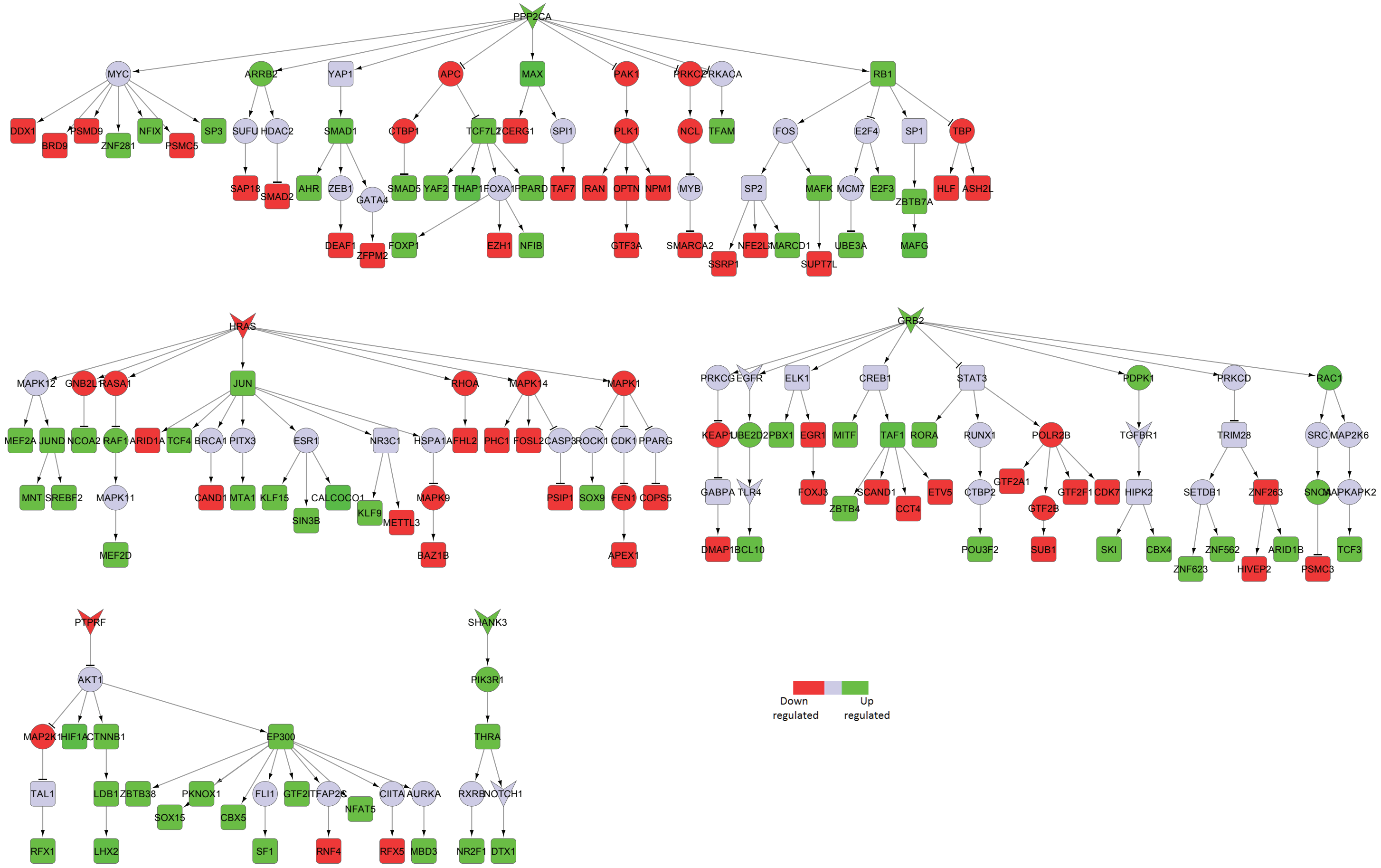

Additional subnetworks identified for hippocampus that did not have known role in AD. The figure legends are the same as that in Fig. 2.

Supplementary Figure 2

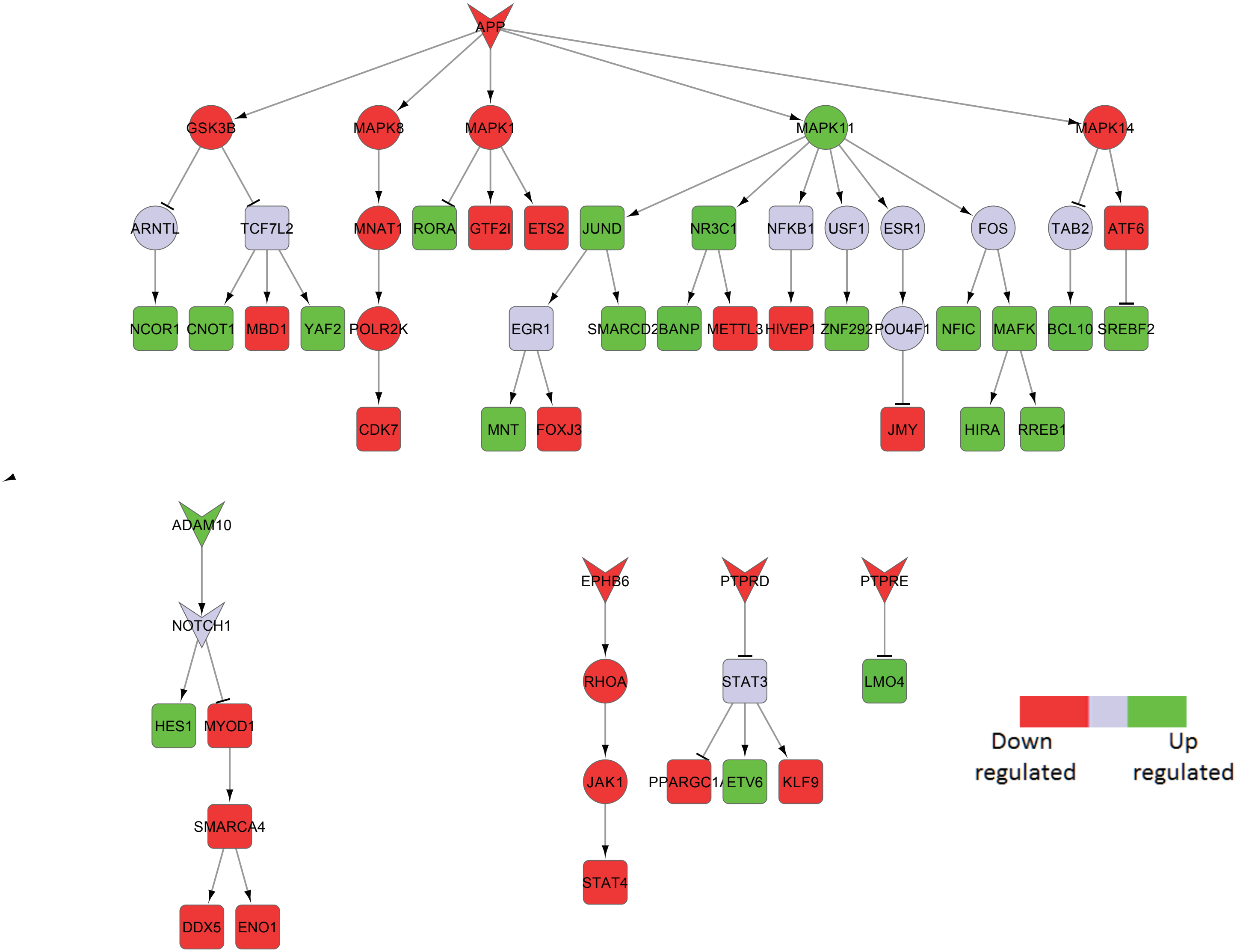

Additional subnetworks identified for posterior cingulate cortex that did not have known role in AD. Figure legends are the same as that in Fig. 2

Supplementary Figure 3

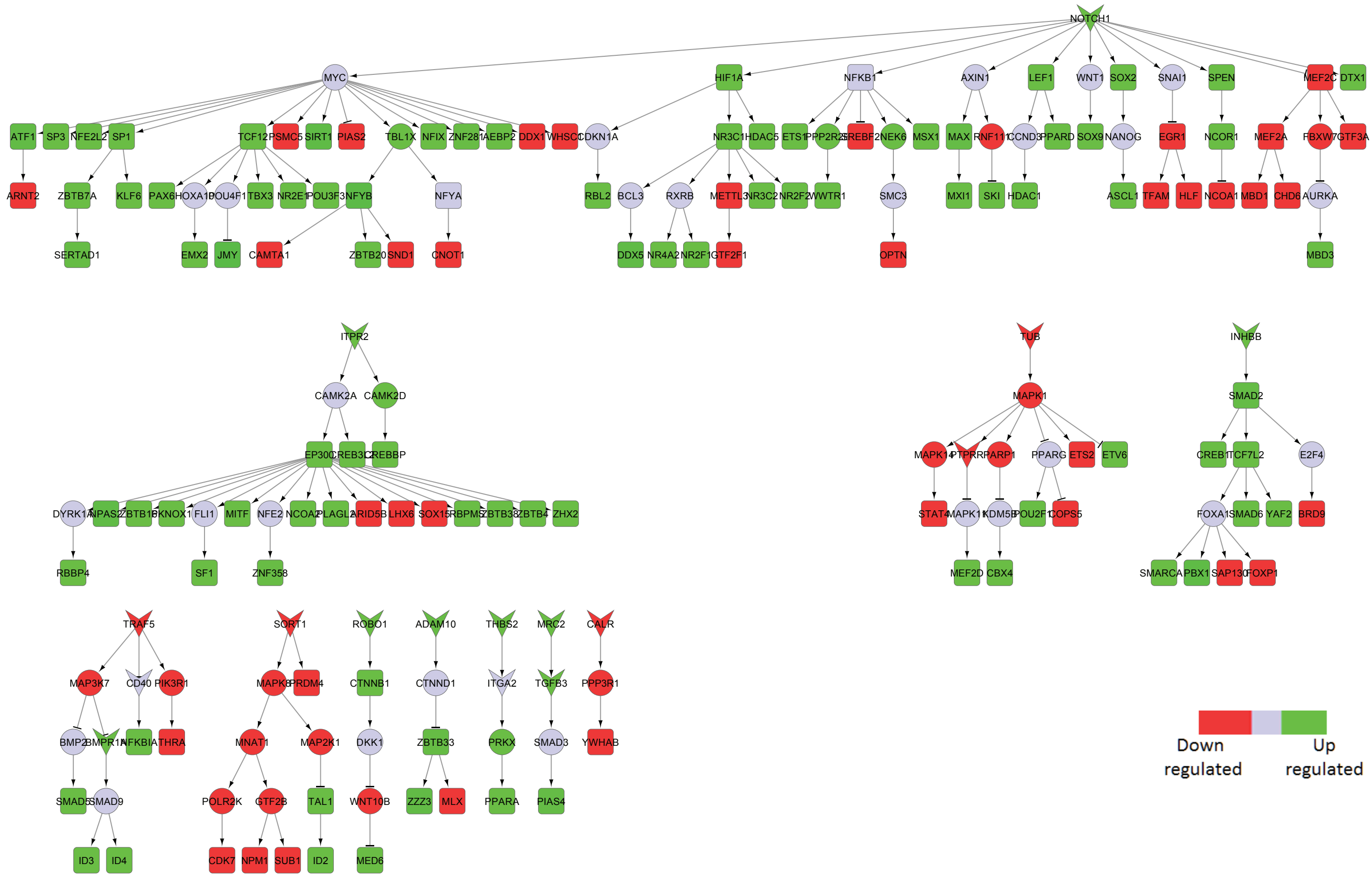

Additional subnetworks identified for middle temporal gyrus that did not have known role in AD. The figure legends are the same as that of Fig. 2

Supplementary Figure 4

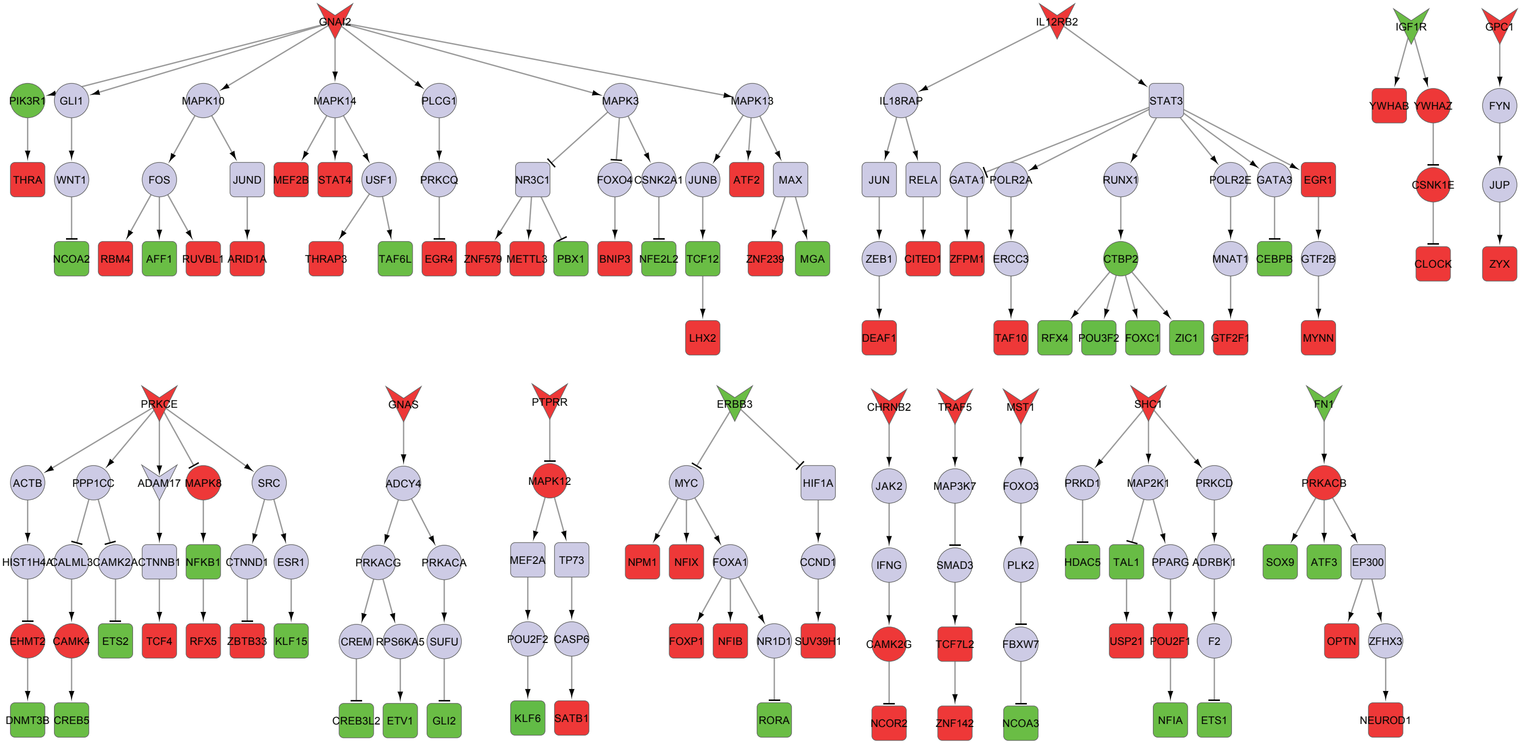

Additional subnetworks identified for entorhinal cortex that did not have known role in AD. The figure legends are the same as that in Fig. 2

Supplementary Figure 5

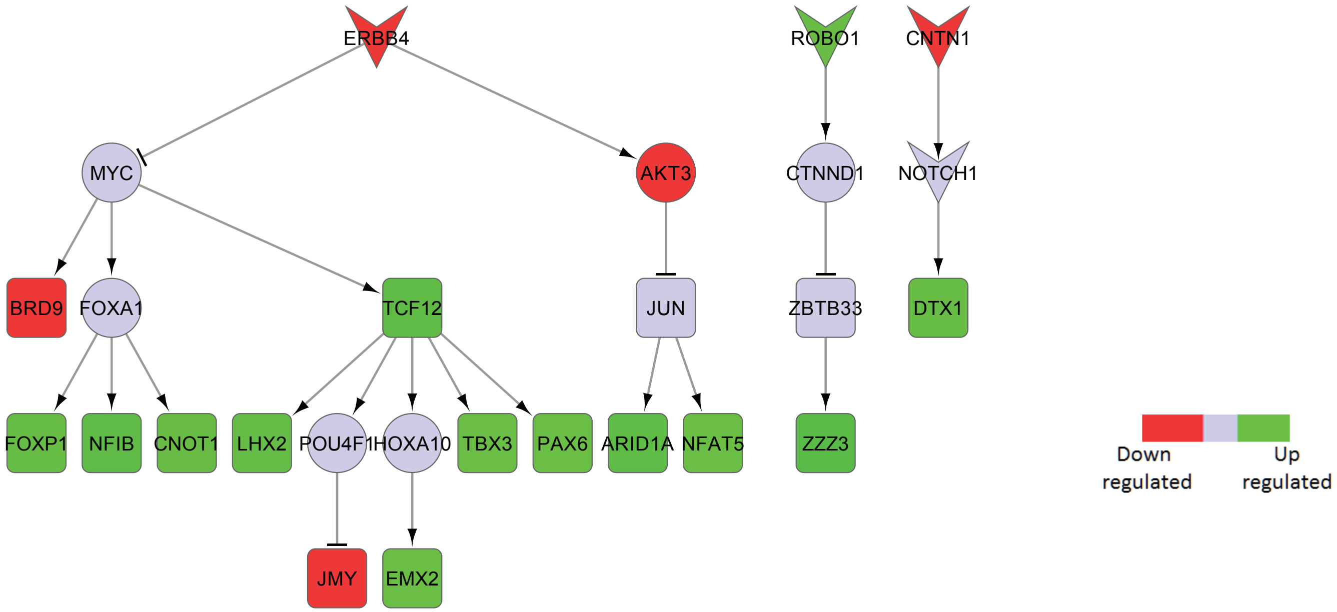

Additional subnetworks identified for primary visual cortex that did not have known role in AD. The figure legends are the same as that in Fig. 2
